# A landscape view on the interplay between EMT and cancer metastasis

**DOI:** 10.1038/s41540-018-0068-x

**Published:** 2018-08-23

**Authors:** Chunhe Li, Gabor Balazsi

**Affiliations:** 1 Shanghai Center for Mathematical Sciences, Fudan University, Shanghai, China; 2 Institute of Science and Technology for Brain-Inspired Intelligence, Fudan University, Shanghai, China; 3 The Louis and Beatrice Laufer Center for Physical and Quantitative Biology, Stony Brook University, Stony Brook, New York, USA; 4 Department of Biomedical Engineering, Stony Brook University, Stony Brook, New York, USA

## Abstract

The epithelial–mesenchymal transition (EMT) is a basic developmental process that converts epithelial cells to mesenchymal cells. Although EMT might promote cancer metastasis, the molecular mechanisms for it remain to be fully clarified. To address this issue, we constructed an EMT-metastasis gene regulatory network model and quantified the potential landscape of cancer metastasis-promoting system computationally. We identified four steady-state attractors on the landscape, which separately characterize anti-metastatic (A), metastatic (M), and two other intermediate (I1 and I2) cell states. The tetrastable landscape and the existence of intermediate states are consistent with recent single-cell measurements. We identified one of the two intermediate states I1 as the EMT state. From a MAP approach, we found that for metastatic progression cells need to first undergo EMT (enter the I1 state), and then become metastatic (switch from the I1 state to the M state). Specifically, for metastatic progression, EMT genes (such as ZEB) should be activated before metastasis genes (such as BACH1). This suggests that temporal order is important for the activation of cellular programs in biological systems, and provides a possible mechanism of EMT-promoting cancer metastasis. To identify possible therapeutic targets from this landscape view, we performed sensitivity analysis for individual molecular factors, and identified optimal interventions for landscape control. We found that minimizing transition actions more effectively identifies optimal combinations of targets that induce transitions between attractors than single-factor sensitivity analysis. Overall, the landscape view not only suggests that intermediate states increase plasticity during cell fate decisions, providing a possible source for tumor heterogeneity that is critically important in metastatic progress, but also provides a way to identify therapeutic targets for preventing cancer progression.

## Gene network: landscape quantifies the interplay between EMT and metastasis

Landscape reveals the underlying mechanisms of the interplay between EMT and cancer metastasis. Professor Chunhe Li at Fudan University and his collaborators constructed an EMT-metastasis gene regulatory network model and quantified the potential landscape of cancer metastasis system. Four attractors emerge on the landscape, characterizing antimetastatic (A), metastatic (M), and two intermediate cell states, respectively. For metastatic progression, cells need to first undergo EMT, and then become metastatic. This suggests that temporal order is critical for the activation of cellular programs in biological systems, and provides a possible mechanism of EMT-promoting cancer metastasis. By optimizing the transition actions between the A and M attractors, the authors identified some optimal combinations of regulatory targets for preventing cancer progression. Combining the landscape with transition action optimization provides a general way to investigate stochastic dynamics of gene regulatory systems.

## Introduction

Cancer metastasis, as the most fatal stage of cancer, accounts for over 90% of cancer deaths.^1^ The epithelial–mesenchymal transition (EMT) plays critical roles in embryonic development and might contribute to cancer metastasis.^2–5^ Many classical EMT marker genes have been connected to cancer metastasis.^6^ However, it remains elusive how to elucidate the mechanistic connections between EMT and cancer metastasis quantitatively.

Mathematical modeling has been useful for studying EMT^7,8^ and metastasis^9^ starting from gene regulatory networks. Yet, in biological systems it is important to consider the effects of the stochasticity, and it remains challenging to study the global properties of the dynamical systems and the gene regulatory networks.^10–14^ For example, the classic Waddington landscape^15^ has been proposed as a metaphor to explain cellular development and differentiation. Recently, the epigenetic landscape for the biological networks has been constructed from different approaches,^16–23^ and employed to investigate the stochastic dynamics of embryonic development and cancer.^13,16,24–34^ In the landscape picture, different cell types are described as the basins of attraction on a potential surface, and cell fate determination is viewed as a ball rolling from one basin to another on the landscape surface by crossing certain potential barriers. The barrier heights separating the attractors or basins quantify the degrees of difficulty for cells to switch from one cell type to the other. Of note, although many papers have been published using landscape approaches for modeling networks of genes and living cells, the theoretical foundation of this approach has only became available very recently, i.e., Waddington’s epigenetic landscape metaphor now has a rigorous mathematical and chemical kinetic foundation.^35–37^ These recent studies show that the landscapes of biochemical reaction networks emerge in the mathematical limit *N*→∞.^35–37^

In this work, we aim to discover the relationship between EMT and metastasis, and study the mechanism of EMT-promoting metastasis from a gene regulatory network perspective. To do so, we first construct an EMT-metastasis regulatory network by merging an EMT gene network and a metastasis network obtained by literature mining, and then develop a computational model corresponding to this joint system. Using this model, we find four steady states (attractors), two of which represent the anti-metastatic (A) and metastatic (M) states, while the other two represent intermediate states (I1 and I2). The intermediate state I1 is similar to the EMT state, with higher expression of the EMT marker genes (such as ZEB). This tetrastable landscape of metastasis is corroborated by recent single-cell experimental data. More interestingly, we find that the cancer metastasis process is a stepwise process, by first stepping into an intermediate state I1 (or EMT state), and then switching to the metastatic state. We propose that the existence of intermediate states increases the plasticity of cancer cells, offering a possible source for tumor heterogeneity. Moreover, we find that the temporal order for the activation of EMT marker genes and metastasis marker genes is critical for metastatic progression. First, the EMT marker gene ZEB is activated, which then promotes the activation of metastatic genes. This suggests a mechanism for EMT-promoting metastasis.

To identify effective anti-cancer strategies from our computational models, we performed single-factor sensitivity analysis to unravel how each link influences the landscape topography. We used the transition action as a measure to quantify the difficulty of transition between attractors, since a smaller transition action, corresponding to a smaller barrier height, means that it is easier for the system to make the corresponding transition. Specifically, we changed each activation or inhibition constant (characterizing individual regulation strength) individually to study their influences on the transition actions between the A attractor and the M attractor. We identified some optimal combinations of targets from minimizing transition actions from the metastatic (M) state to the anti-metastatic (A) state. We show quantitatively that the strategies from our optimization method are more effective than those from the single-parameter sensitivity analysis. This supports the view that advanced cancer is a network disease rather than a disease arising from single-gene defects. Therefore, an effective therapeutic strategy should be targeting multiple networks with different functions (e.g., related to different hallmarks of cancers) in appropriate order. Our results suggest that intermediate states play critical roles in cancer progression and offer a possible explanation for tumor heterogeneity. We also provide an approach to identify the optimal combinations of therapeutic targets for preventing cancer progression.

## Results

### Mathematical model for the EMT-metastasis network

**Fig. 1:**
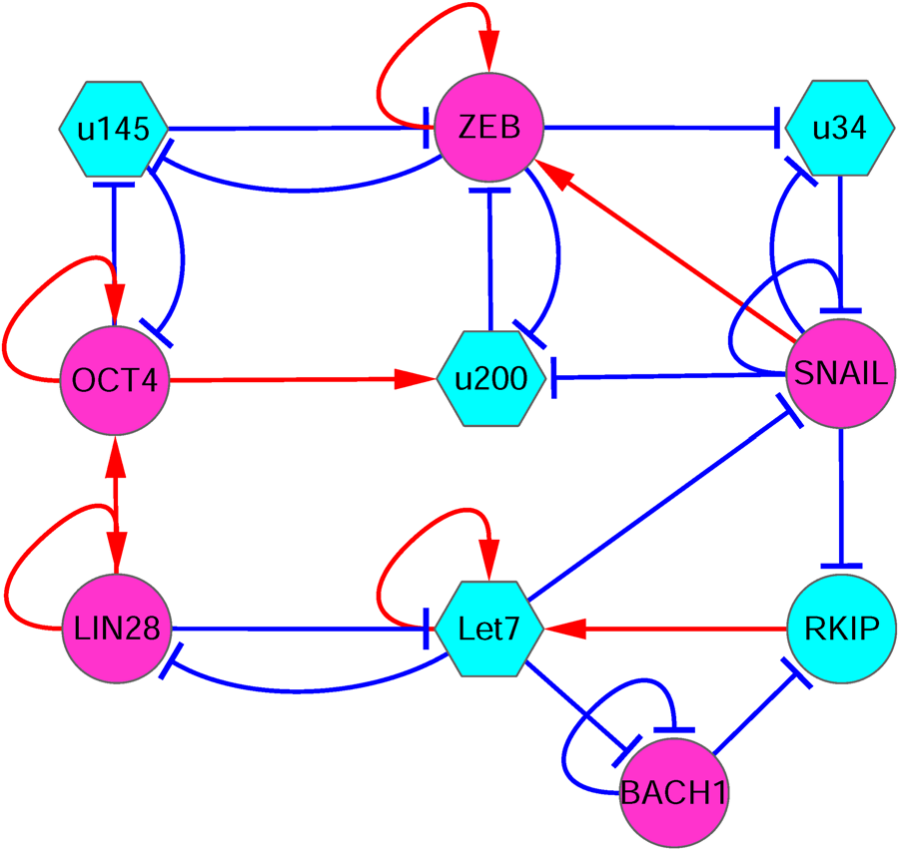
The diagram for the core circuit of EMT-metastasis network including 10 gene nodes and 26 regulation links (8 activations and 18 repressions). Red arrows represent activation and blue bars represent repression. Magenta nodes represents pro-metastatic genes, and cyan nodes represent anti-metastatic genes. Circle nodes represent proteins and hexagonal nodes represent microRNAs. u34: miR34, u200: miR200, u145: miR145

Based on previous computational works on EMT and cancer metastasis,^9,38^ we first constructed an EMT-metastasis gene regulatory network by literature mining (Fig. 1), which includes 10 nodes (genes) and 26 links (regulations). Based on the network structure, we generated the ordinary differential equations (ODEs) describing the time evolution of relative expression levels for each of the 10 genes. To incorporate biological knowledge to the model, we developed these equations based on the following:

1. The mathematical equations for the let7–RKIP–BACH1 circuit originate from the model in ref. ^9^ and include biological details such as the interaction between let7 and BACH1. Hill coefficients for the regulations among let7, RKIP, and BACH1 were likewise taken from ref. ^9^.
2. For the EMT and stemness gene regulatory circuit, our models are based on other recent studies.^38,39^ The numbers of binding sites for the interaction between microRNAs and proteins constrain the choice of Hill coefficients (see Table S1 in SI).
3. For finding the other parameter, a major biological constraint is to obtain multistability because in a cancer metastasis system there should be at least two cell states, i.e., the metastatic cell state and the anti-metastatic cell state.

We used Hill functions to describe the activation and inhibition regulations among different genes.^16,21,24^ The ODEs that govern the time evolution of variables (relative expression level of different genes) are in Eq. (1):

1 $$F_i = \mathop {\sum}\limits_{j = 1}^N \frac{{A_{ji}X_j^n}}{{S^n + X_j^n}} + \mathop {\sum}\limits_{j = 1}^N \frac{{B_{ji}S^n}}{{S^n + X_j^n}} - kX_i$$

and the ODEs for let7 (*X*_7_) and BACH1 (*X*_10_) are^9^:

2 $$F_7 = \frac{{sa2X_7^{ns}}}{{S^{ns} + X_7^{ns}}} + \frac{{G_LX_8^{nr}}}{{S_R^{nr} + X_8^{nr}}} + \frac{{bS^n}}{{S^n + X_9^n}} - k_{LB}X_7X_{10} - kX_7$$

3 $$F_{10} = G0_B + \frac{{G_BS_B^{nb}}}{{S_B^{nb} + X_{10}^{nb}}} - k_{LB}X_7X_{10} - kX_{10}.$$

Here, *F*_*i*_(*i* = 1, 2, ..., 10) represents the driving force for the time evolution of expression levels of 10 genes. There are totally *N* = 10 equations describing the time evolution of 10 variables. *S* represents the threshold of the sigmoidal function, and *n* is the Hill coefficient, which determines the steepness of the sigmoidal function.^17,24^ Here, the default parameter values for the Hill function are specified as: *S* = 0.5, *n* = 4. *A* and *B* are the interaction matrices for activation and inhibition interactions. *A*_*ji*_ = *a* when gene *j* activates gene *i*, otherwise *A*_*ji*_ = 0. *B*_*ji*_ = *b* when gene *j* inhibits gene *i*, otherwise *B*_*ji*_ = 0 (see Table S2 for the interaction matrix *M*). In addition, *a* is the activation constant, *b* is the inhibition constant, and *k* is the degradation rate for different genes (see the Supporting Information for the descriptions of parameters, and Table S1 for the values of parameters). In Eq. (1), the first term represents the activation effect of gene *j* on gene *i* (this term represents the self-activation when *i* = *j*), while the second term represents the repression effect from gene *j* to gene *i* (this term represents the self-repression when *i* = *j*). Finally, the last term represents the degradation of gene products (proteins, RNAs).

### Tetrastable landscape for cancer metastasis

**Fig. 2:**
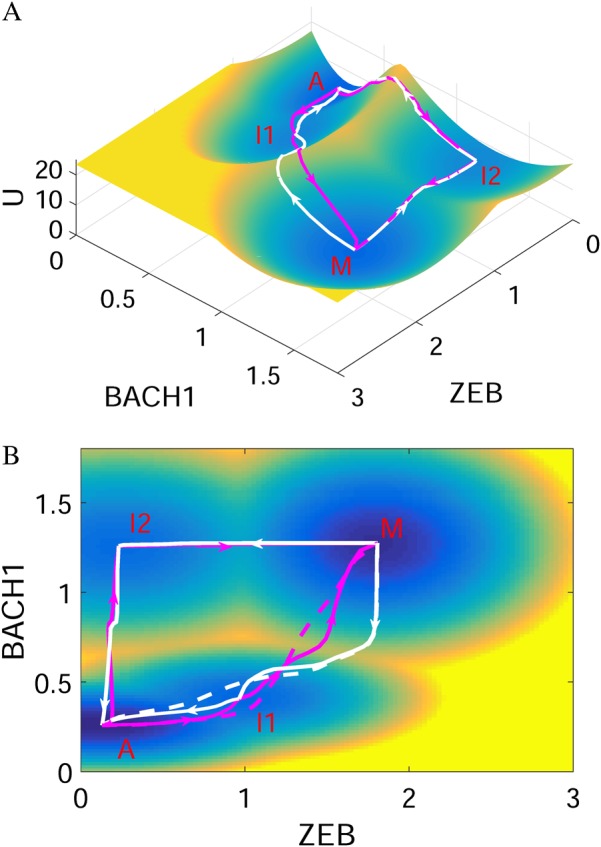
The landscape and corresponding minimum action paths (MAPs) for the EMT-metastasis network are shown in three-dimensional **(a)** and two-dimensional figures **(b)**. Magenta solid lines represent the MAP from A state (anti-metastatic cell state) to I states, and to M state (metastatic cell state), and the white solid lines represent the MAP from M to I, and to A state. The dashed lines represent the direct MAP from A to M and from M to A states, respectively. A: anti-metastatic state, M: metastatic state, I1, I2: intermediate state. Here, ZEB and BACH1 are selected as the two coordinates

The probabilistic evolution for a stochastic dynamical system is governed by the diffusion equations. For a high-dimensional gene regulatory system such as the one here, it is difficult to solve the diffusion equations directly. Following the self-consistent approximation approach (see Methods), we determined the steady-state probability distribution and then mapped out the potential landscape for the EMT-metastasis system. Because it is difficult to visualize the landscape in a ten-dimensional space, we selected two variables as the coordinates and projected the ten-dimensional landscape into this two-dimensional space, by integrating out the other eight gene variables. We chose the two key variables “ZEB” and “BACH1” as the two coordinates for the landscape, since ZEB is a major EMT marker gene, and BACH1 is a major metastasis marker and regulator gene. We need to stress that our major conclusions do not depend on the specific choice of the coordinates (see Figs. S1–S4 for landscapes with other gene pairs as coordinates) because we also calculated the transition actions between different attractors, based on the ten-dimensional space of gene expressions. We found four stable cell states emerging on the landscape for the EMT-metastasis system (Fig. 2). The landscape surface is characterized by different colors, where the blue region represents lower potential or higher probability, and the red region represents higher potential or lower probability. The four basins of attraction on the landscape represent four different cell states characterized by different gene expression patterns in the ten-dimensional state space. These states separately correspond to M state (metastatic state, high ZEB/high BACH1 expression), A state (anti-metastatic state, low ZEB/low BACH1 expression), and two intermediate states (I1 and I2, intermediate ZEB and BACH1 expression).

Of note, besides for the anti-metastatic state (A state) and metastatic state (M state), the two other intermediate states (I1 and I2) appear on the landscape. We propose that the I1 state corresponds to the EMT/partial EMT or some similar state,^7,8^ since it has higher expression of EMT marker genes (such as ZEB and SNAIL), but lower expression of metastatic marker genes (such as BACH1).

### Metastasis is a stepwise process

To study the transitions among individual cell types, we calculated kinetic transition paths by minimizing the transition actions between attractors,^31,40^ obtaining minimum action paths (MAPs). The MAPs for different transitions are shown on the landscape in Fig. 2. The magenta MAP from the A state to the M state corresponds to the pro-metastatic process, while the white MAP from the M state to the A state corresponds to anti-metastatic process. The lines represent the MAPs, with the arrows denoting the directions of the transitions. The MAP for pro-metastatic process and the MAP for anti-metastatic process are irreversible, since the forward and reverse kinetic paths are not identical. This irreversibility of kinetic transition paths is caused by the non-gradient force, or curl flux.^14,20,41^

**Fig. 3:**
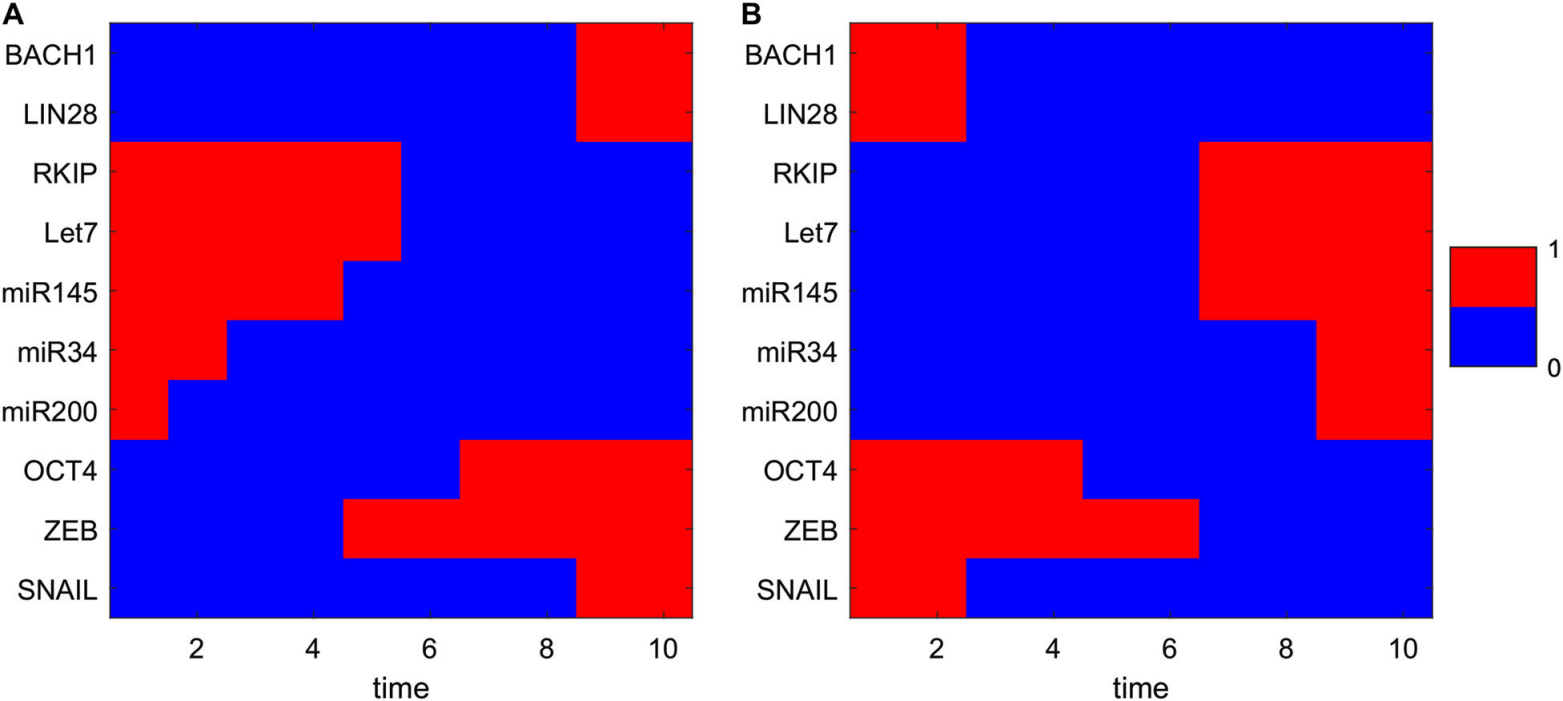
Transition path from A state to M state (**a** corresponding to the metastatic progression) and from M state to A state (**b** corresponding to the anti-metastatic progress) in terms of expression levels of 10 different genes. The relative gene expressions are discretized to 0 or 1. 1 represents that the corresponding genes are in the on (activated) state and 0 represents that the corresponding genes are in the off (repressed) state. *X*-axis shows the 10 time points along the transition path

Since there are two intermediate states between A and M, in principle there should be two routes from the A to the M attractor, i.e., passing through the I1 state or passing through the I2 state. However, by calculating the MAP from the A to the M state, we found that the MAP from A to M and the MAP from M to A (dashed lines in Fig. 2b) are more similar to the case of passing through the I1 state, compared to that passing through I2 state (Figs. 2, 3). This demonstrates that for the transition process from A to M, i.e., metastatic progression, the cells experience a staged transition process. Specifically, cells first rise along the ZEB axis reaching the I1 state (EMT state), and then move up along the BACH1 axis, which possibly corresponds to a process in which cells acquire increased ability of invasion. This suggests that the EMT state might serve as a premetastatic state, promoting the progression toward metastasis. For the reverse process, i.e., the transition from metastatic M state to anti-metastatic A state, cells first decrease the expression of BACH1 reaching the I1 state and then decrease ZEB expression, finally reaching the A state. This indicates that a better anti-metastasis strategy would be first targeting BACH1 inducing the transition from M to I1, rather than first targeting ZEB governing the EMT process.

To investigate the metastatic process for multiple gene expressions, we visualized the ten-dimensional MAP from the A to the M state by discretizing the expressions of the 10 genes. From Fig. 3, we found that for the pro-metastatic process, three microRNAs (miR145, miR34, miR200) need to be downregulated first, and then the EMT marker gene ZEB must be activated, leading to the activation of the metastasis gene BACH1. These results indicate the importance of microRNAs in preventing cells from obtaining metastatic ability. Additionally, ZEB is activated before BACH1 (consistent with the 2D landscape picture in Fig. 2). This suggests that the order of switching on or switching off for different genes is critical and cells need to undergo EMT before transforming into metastasis cells.

**Fig. 4:**
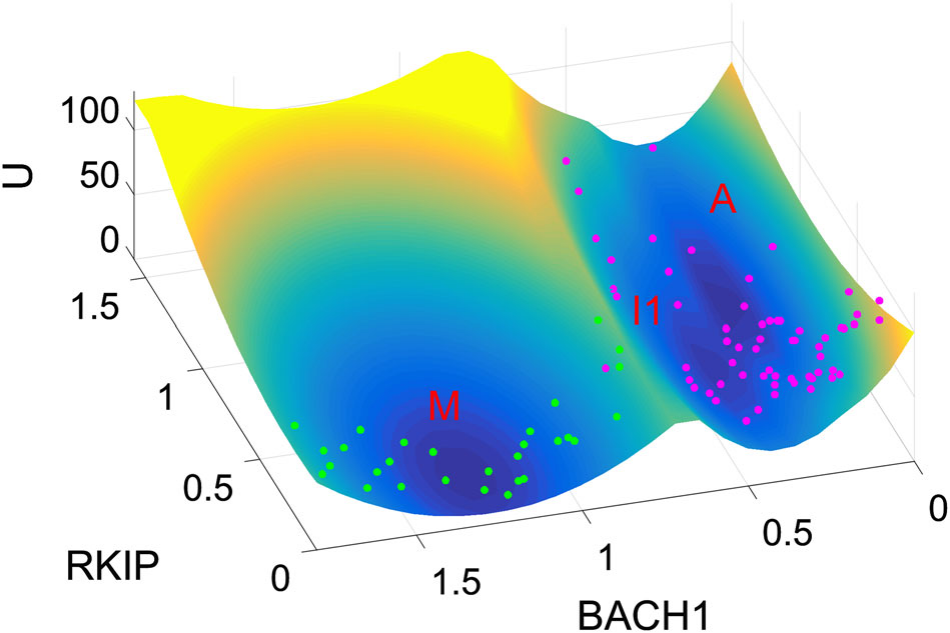
Landscape comparisons with single-cell experimental data (MDA-MB-231 cells). RKIP and BACH1 are chosen as the two coordinates. Each point represents a normalized gene expression value for one cell from single-cell experiments.^9^ Green points, untreated cells; magenta points, cells after shBACH1 treatment (BACH1 knockdown). M: metastatic state, A: anti-metastatic state, I1: intermediate state

Our landscape picture is also supported by the recent single-cell experiments, considering the expression of BACH1 and RKIP (Fig. 4),^9^ mapped to the landscape with BACH1 and RKIP as the two coordinates. Green points (each point represents a single cell) correspond to untreated breast cancer cells, while magenta points correspond to breast cancer cells after shBACH1 treatment (BACH1 knockdown). We find that the untreated metastatic cells are located around the metastasis (M) basin, whereas after BACH1 knockdown treatment, cells relocate around the A and I1 basins. This means that BACH1 knockdown lowers the energy barrier between the M basin and I1 basin, promoting the transition from the M state to the I1/A state. There are also some cells around the saddle point (green points), which reflects the heterogeneity of cancer cells and knockdown efficiency. Indeed, the single-cell data indicates that not all cells move to the A state. Yet, both A state cells and I1 state cells are separated from the M state, suggesting efficient treatment, because both A state cells and I1 state cells lose their metastatic ability (losing their expression of BACH1). Therefore, displaying these single-cell data on the landscape supports the existence of the intermediate state in the metastatic progression. It also indicates that a possible strategy for the metastasis prevention is to push back cells into the intermediate I1 state (or EMT state). This also means that an effective way of metastasis prevention would be to target an EMT cell before it enters metastatic state, since the I1 state or EMT state is much easier to convert into the anti-metastatic A state compared with the M state (Figs. 2, 4).

### Sensitivity analysis for individual links

Traditionally, cancer has been studied as a disease arising from individual genetic defects (a disease from individual mutation). Recently, however, it has been proposed that cancer is a network diseases,^42–44^ which can be understood as attractors in the state space of gene regulatory networks.^42,45–47^ Therefore, it is important to ask how to alter normal (or anti-metastatic) and cancer (or metastatic) attractors to stabilize normal attractors and destabilize cancer attractors by targeting certain genes or regulations between genes. In the gene network models, this corresponds to the task of identifying the critical parameters that determine the metastatic to anti-metastatic state transitions. One natural way to accomplish this is to perform a single-factor sensitivity analysis for barrier heights between the M attractor and the A attractor, or the transition actions from M attractor to A attractor. This is preferable since a multiple-factor sensitivity analysis has very large computational cost.

Here we used two quantities to measure the difficulty of transitions between attractors. To quantify the stability of the system from the landscape topography, we defined the potential barrier height as the potential difference between a local minimum and the corresponding saddle point. A larger barrier means that it is harder for the system to surmount the barrier and switch from one attractor to another. Therefore, the barrier height provides a quantitative measure for the relative stability of each attractor and measures the difficulty of transitions among attractors in the system. Additionally, we also used transition actions to quantify the difficulty of transitions between attractors, since a smaller transition action, corresponding to a larger potential barrier, means an easier transition between attractors. In principle, the transition action should be correlated with the potential barrier height. However, we obtained the barrier height from the self-consistent and Gaussian approximation, so the transition action provides a more precise description for the barrier-crossing process.

**Fig. 5:**
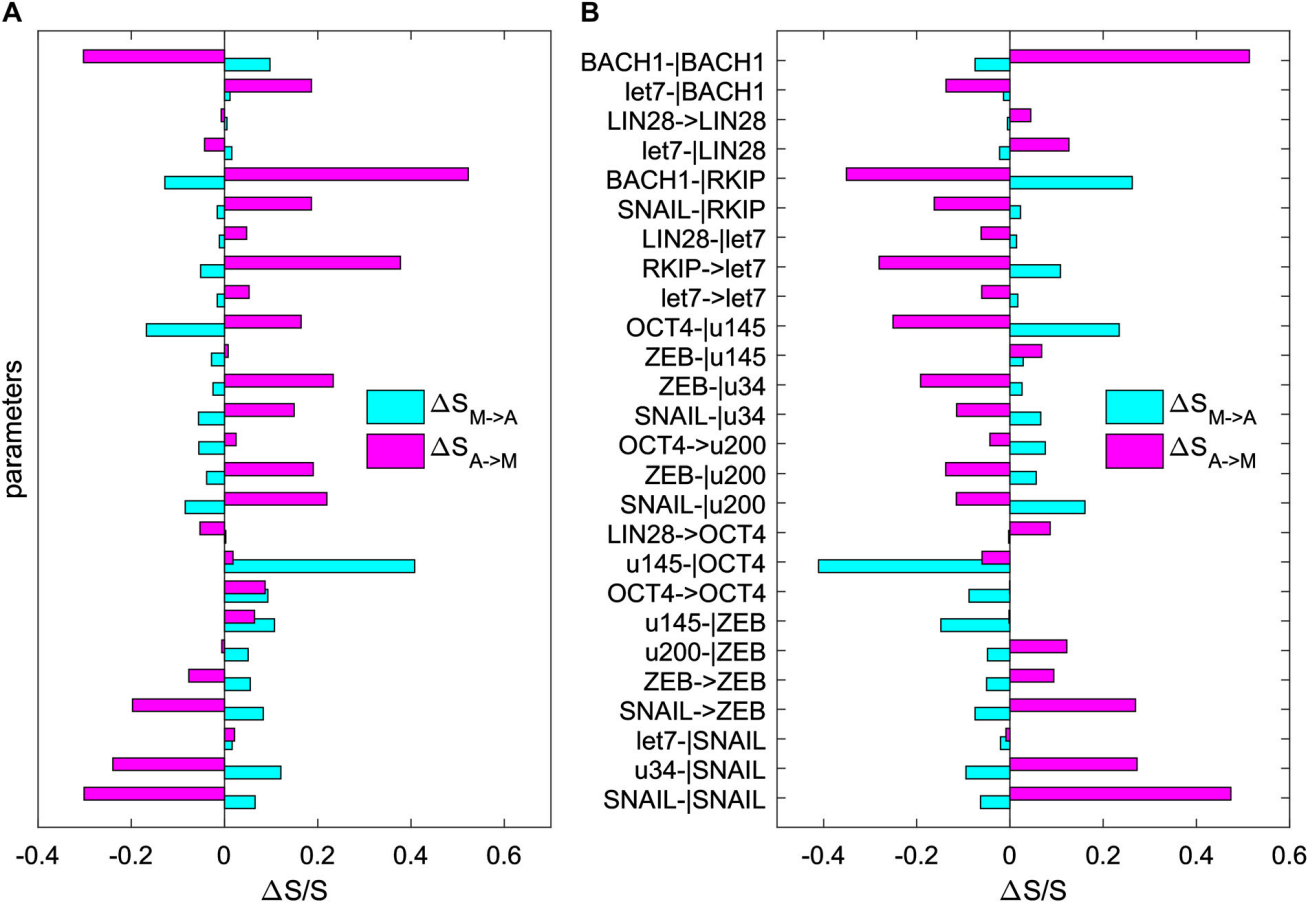
Sensitivity analysis for the 26 key parameters (regulatory strengths among different genes, including 8 activation constants and 18 inhibition constants) on the transition action (*S*_*M*−>*A*_ and *S*_*A*−>*M*_). *Y*-axis represents the 26 parameters. *X*-axis represents the percentage of the change of the transition action (S) relative to S with default parameters. Here, *S*_*M*−>*A*_ represents the transition action from attractor M to attractor A (cyan bars), and *S*_*A*−>*M*_ represents the transition action from attractor A to attractor M (magenta bars). **a** Each parameter is increased by 20%, individually. **b** Each parameter is decreased by 20%, individually. u145: miR145, u34: miR34, u200: miR200

Our sensitivity analysis is based on calculating the change of the transition actions between attractors after changing each parameters. Specifically, we change each activation or repression constant (representing individual regulation strength) individually to see how the transition actions between the M attractor and the A attractor are affected. In this way, we can identify those critical elements (here we focus on the links between genes) that govern the M to A transitions, as shown in Fig. 5. We find that some paramount links (to change the transition actions between M and A attractor significantly) include (see Table S3 for link ID): the self-repression of BACH1 (link 26), the repression of BACH1 on RKIP (link 22), the activation of RKIP on let7 (link 19), the repression of OCT4 on miR145 (link 17), the repression of miR145 on OCT4 (link 9), the activation of SNAIL on ZEB (link 4), the repression of miR34 on SNAIL (link 2), the self-repression of SNAIL (link 1). These key regulations are sorted according to their sensitivity (defined as the relative change in transition actions caused by each parameter change). The top 10 sensitive regulations are shown in Table S4.

The predictions from this sensitivity analysis agree well with experimental data. For example, experiments show that Snail plays a critical role in tumor growth and metastasis of ovarian carcinoma through regulation of MMP activity,^48^ ZEB and Snail are upregulated in metastatic cells,^4^ and the transcription factor BACH1 has been identified as a regulator of metastasis-associated genes, which are critical for bone metastasis formation.^9,49^ Additionally, the interaction between the microRNA miR145 and the stem cell pluripotency gene OCT4 might play important roles in tumor growth and invasion,^50^ and the microRNA let7 is proposed to inhibit cell motility by regulating the genes in the actin cytoskeleton pathway in breast cancer.^51^ The consistency between our model predictions and previous experiments supports our models and landscape approaches.

### Landscape control identifies optimal combinations of therapeutic targets

The single-factor sensitivity can be used to discover the effects of individual genes on the landscape or the transition actions. However, the combined influence from multiple parameters is difficult to identify in this way because it requires multiple-factor sensitivity analysis on parameters, with a large computational cost. Based on transition actions optimization,^31,52,53^ we can predict optimal therapeutic combinations for targeting multiple genes or regulations among them for controlling the landscape. Therefore, we aimed to identity the optimal combinations of targets (regulations) for inducing M to A state transitions by optimizing (minimizing) the transition action from the M to the A attractor.

The top 10 targets from optimization are shown in Table 1. The comparison between Table 1 and Fig. 5 (or Table S4) indicates good consistency, in addition to some differences. For example, considering the fact that experimentally it might be only possible to tune a few genes (e.g., three) simultaneously, we found that the top three targets from the MAP optimization approach (Table 1, link 22, 17, 26) are not identical to the top three targets from the single-factor sensitivity analysis (Table S4, link 9, 17, 22). Yet, the both strategies suggest concordantly that an efficient treatment should target multiple circuits with different functions simultaneously. For example, link 22 (repression of BACH1 on RKIP) and link 17 (repression of OCT4 on miR145) separately characterize the metastasis circuit and the EMT circuit. Nonetheless, the optimization results suggest a better strategy (by adding a third interventions), namely, to target link 26 rather than link 9 for a larger sensitivity. This is probably because link 9 shares some of its function with link 17 since they are both characterizing the mutual repression between OCT4 and miR145. Therefore, a better strategy is to target another key metastasis gene BACH1 (link 26) rather than the repression of miR145 on OCT4 (link 9). This result is reasonable, and offers a quantitative illustration about why the MAP optimization method is useful to identify successful interventions, which would be difficult to identify from single-factor sensitivity analysis.

**Table 1:** Metastasis-suppressing interventions identified from optimization of transition actions for EMT-metastasis model

Target ID	Direction	Biological meaning of intervention	Sensitivity	Supporting references
22	↓	Decrease repression of BACH1 on RKIP	27.9%	Prediction
17	↓	Decrease repression of OCT4 on miR145	7.0%	^50^
26	↑	Increase self-repression of BACH1	7.0%	^9, 49^
19	↓	Decrease activation of RKIP on Let7	6.7%	^51^
25	↑	Increase repression of Let7 on BACH1	6.1%	^9, 49^
2	↑	Increase repression of miR34 on SNAIL	6.0%	^4, 48^
1	↑	Increase self-repression of SNAIL	5.8%	^48^
15	↓	Decrease repression of ZEB on miR34	4.5%	Prediction
13	↑	Increase activation of OCT4 on miR200	4.3%	Prediction
20	↓	Decrease repression of LIN28 on Let7	3.2%	^51^

Each regulation is represented by a target ID (Table S3). Direction represents the direction of changes in regulation strengths for the corresponding interventions. Sensitivity is defined as how much each parameter (regulation strength) change accounts for the total change of parameters, averaged across the six parameter sets (representing the heterogeneity of tumor samples). Here, the targets are sorted by sensitivity and the top 10 targets are showed. The top 10 parameter changes account for about the 79% of the total parameter change. The column of “Supporting references” shows the consistent experimental evidences for the corresponding interventions

**Fig. 6:**
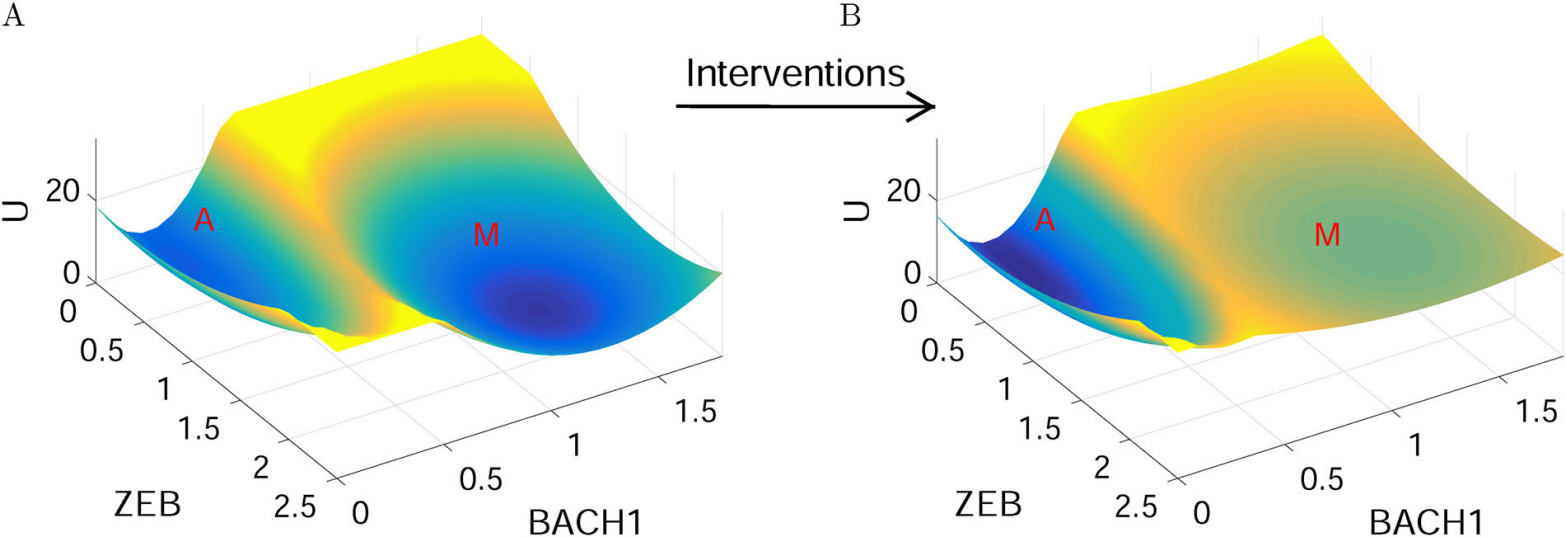
An illustration for the landscapes of cancer metastasis network before and after interventions. Before interventions, the M state is deep and stable; after interventions, the M state becomes very shallow and cells are more inclined to stay in the A state. A: anti-metastatic state, M: metastatic state

Finally, we can infer how the landscape of the metastasis network looks before and after interventions suggested from above procedures (Fig. 6). Before any interventions, the M state is deep and stable; after interventions, the M state becomes very shallow and cells are more inclined to remain in the A state, suggesting a successful intervention for cancer metastasis treatment. Similar results could also provide an explanation for the relapse of cancers after treatment. A potential reason could be that many drugs for tumors are only pushing cells away from cancer attractors (changing the populations of tumor cells), while leaving the cancer metastasis network landscape unaltered. This again underlines that cancer cells are characterized by the attractors determined by cancer networks, and an effective way for cancer treatment should not be only killing tumor cells (i.e., not changing the landscape topography), but rather changing the landscape of cancer networks (making the cancer or metastatic attractors less stable as illustrated in Fig. 6).^28^ This could be implemented by targeting multiple genes or regulations simultaneously as suggested in this work.

## Discussion

Understanding the mechanisms underlying metastatic progression is critical to developing new therapeutic strategies for cancer treatment. Accumulating evidences show that stochastic fluctuations play important roles in cancer progression.^54^ EMT is considered to be vital to the cancer metastatic process. However, the molecular mechanisms for how EMT promotes metastasis are not fully understood. In this work, based on the stochastic dynamics theory of gene networks we revealed the EMT-metastasis landscape through constructing an EMT-metastasis gene regulatory network. We identified four stable cell states characterized by the attractors on the landscape, in agreement with recent single-cell experimental data. Besides for the anti-metastatic attractor and metastatic attractor, we found two intermediate states (I1 and I2), one of which is very similar in gene expression pattern to an EMT cell state. This implies that metastatic progression is a stepwise process and there are two possible routes for the A to M transition, i.e., going through the I1 or going through the I2 state. However, our results based on MAPs between attractors indicate that the MAP for the A to M transition is closer to the path from A to I1, and then to M, i.e., the metastatic progression prefers going through the I1 (EMT) state rather than the I2 state.

These landscape results have a number of implications for cancer progression. First, cancer metastasis is a stepwise process. For the metastatic progression, the first step for cells is to cross the barrier between A and I1 and enter the I1 or EMT state (meaning that cells finish the EMT transition). The second step is crossing the barrier between I1 and M state to enter the metastatic state. The staged process for metastatic progression or the existence of intermediate states increases the plasticity of cell fate determinations. It also provides a possible source for tumor heterogeneity. Second, the intermediate state I1 provides a possible therapeutic anti-cancer target because it would be much easier to induce metastatic cells switching to the I1 state than switching to the A state. Third, our results indicate that the temporal order is critical for the metastatic progression. EMT circuit (e.g., the EMT marker gene ZEB) has to be activated before the metastatic marker gene BACH1 is activated (Fig. 2). The alternative case would be first activating BACH1 and then activating the EMT circuit (A to I2 and to M), but our transition action results suggest that this route has larger cost (action) and is not preferred by metastatic cells.

Our landscape results agree with recent single-cell data, which disperse within the A, I1, and M basins, as well as around the saddle points between basins, reflecting the heterogeneity of tumor cell populations. Recently, single-cell approaches have been developed to extract gene expression signatures for different cell populations.^55,56^ In principle, one can integrate single-cell data considering more genes involved in EMT, metastasis, as well as other hallmarks of cancer. It would be interesting to combine gene network modeling and such single-cell data analysis, which is still very challenging. This work provides a starting point and a possible way toward that goal.

In the development of novel cancer therapeutic approaches, a major challenge in the field is, can we shift the goal from inducing tumor cell death (i.e., removing cells from particular “basins”) toward a strategy that seeks to alter the landscape topography to prevent relapse. Given the inevitability of cells eventually returning to steady-state proportions following a perturbation or drug treatment, this challenge is significant and important. In this work, we performed some initial attempts to tackle this challenge. We propose some optimal therapeutic targets for cancer treatment, focusing mostly on the regulatory interactions between different genes. We show that the optimal combinations of targets from minimizing transition actions are more effective than the strategy from single-factor sensitivity analysis. These results provide some new insights into potential therapeutic applications by targeting multiple genes. The successful application of these interventions will depend on whether physicians can actually tune these regulations in practice. Recent work indicates that one can tune the strengths of gene regulatory interactions through a synthetic biology approach.^57^ With the further development of experimental techniques, we believe these predictions on optimal interventions of anti-cancer targets can eventually be tested from experimentally, potentially providing the guidance for designing the new anti-cancer strategies.

In this work, for simplicity we only consider the interplay between EMT and cancer metastasis. As suggested by Weinberg et al.,^43,44^ cancer is a complex disease determined by many hallmarks of cancer. It would be really interesting to incorporate the other hallmarks of cancer, such as cancer immunity, cancer metabolism, etc., into our current metastatic network, and rediscover the landscape of cancer.^27^ One limitation of the current work is that we only used single-cell data on two gene products (proteins) for comparisons with our computational model. In principle, one could collect single-cell data involving more EMT-cancer metastasis gene products at the RNA level by single-cell sequencing or at the protein level by mass cytometry. However, these technologies are still not routinely available or affordable to most laboratories, and hence it remains challenging to obtain and combine single-cell data with mathematical modeling. Another limitation of this work is that the current models are single cell based. The cell–cell communications and interactions in populations of cells have been suggested to play critical roles in all kinds of systems such as neuron synchronization in circadian rhythms^58^ and different forms of cancers.^59,60^ Future work could incorporate cell–cell interactions on top of the current gene regulatory network models, where we could study the influence of other factors (e.g., the growh factor secretion and the proliferation rates for different cell types) on the landscape. This could better reflect the underlying biology and allow us to obtain more insights into intricate mechanisms for EMT and cancer metastasis.

## Methods

### Self-consistent mean field approximation

The time evolution a dynamical system is determined by a probabilistic diffusion equation. Given the system state *P*(*X*_1_, *X*_2_, ..., *X*_*N*_, *t*), where *X*_1_, *X*_2_, ..., *X*_*N*_ represent the concentration of molecules or gene expression levels, we expect to have N-dimensional partial differential equation, which are hard to solve because of the very large state space of the system. Following a self-consistent mean field approach,^20,24,41,61,62^ we can split the probability into the products of the individual probabilities: $P\left( {X_1,X_2,...,X_N,t} \right)\sim \mathop {\prod}\nolimits_i^n P_i\left( {X_i,t} \right)$ and solve the probability self-consistently. In this way, we effectively reduce the dimensionality of the system from *M*^*N*^ to *MN*, and therefore the computation of the problem becomes tractable. In some sense, our approximation is similar to the Poisson product result, which was shown to be exact for deficiency zero reaction networks.^63^

However, for the multidimensional system, it is still challenging to solve the diffusion equations directly. We start from the moment equations and assume specific probability distribution based on physical argument, i.e., we assume some specific connections between moments. In principle, once we know all moments, we can obtain the probability distributions. In this work, we assume Gaussian distribution as an approximation, which means we need to calculate two moments, the mean and the variance.

When the diffusion coefficient *D* is small, the moment equations can be approximated to^64,65^:

4 $$\begin{array}{*{20}{c}} . \cr {\overline {\mathbf{x}} } \cr {} \end{array}\left( t \right) = F\left[ {\overline {\mathbf{x}} \left( {\mathbf{t}} \right)} \right]$$

5 $$\mathop {{\mathbf{\sigma }}}\limits^ \cdot \left( t \right) = {\mathbf{\sigma }}\left( t \right){\mathbf{A}}^{\mathbf{T}}\left( t \right) + {\mathbf{A}}\left( t \right){\mathbf{\sigma }}\left( t \right) + 2{\mathbf{D}}\left[ {\overline {\mathbf{x}} \left( {\mathbf{t}} \right)} \right].$$

Here, **x**, ***σ***(*t*), and **A**(*t*) are vectors and tensors, and **A**^**T**^(*t*) is the transpose of **A**(*t*). The elements of matrix *A* are specified as: $A_{ij} = \frac{{\partial F_i\left[ {X\left( t \right)} \right]}}{{\partial x_j\left( t \right)}}$. In this work we model intracellular biochemistry based on stochastic chemical kinetics, since an arbitrary noise structure would not be very realistic. Of note, one fundamental insight from the recent theory is that the landscape is sensitively dependent on the noise structure, e.g., the *A* matrix.^35^ Also, the approach we used here is neither van Kampen Ω-expansion because our drift term F is not linearized, nor the Kramers–Moyal expansion, which is questionable for computing barrier crossing.^66^ It is worth pointing out that a “Large Deviation Theory” of the general stochastic chemical kinetic system has been derived recently by Ge and Qian.^35^

Based on these equations, we can solve **x**(*t*) and ***σ***(*t*). Here, we only consider the diagonal elements of ***σ***(*t*) from the mean field approximation. Therefore, the evolution of probability distribution for each variable can be acquired from the Gaussian approximation:

6 $$P\left( {x,t} \right) = \frac{1}{{\sqrt {2\pi \sigma \left( t \right)} }}{\mathrm {exp}} - \frac{{\left[ {x - \overline x \left( t \right)} \right]^2}}{{2\sigma \left( t \right)}}.$$

Here, $\overline {\bf{x}} ({\it{t}})$ and ***σ***(*t*) are the solutions of Eqs. (2) and (3). The probability distribution obtained above corresponds to one steady state or basin of attraction. If the system has multiple steady states, then there should be several probability distributions localized at each basin with different variances. Therefore, the total probability is the weighted sum of all these probability distributions. The weighting factors (*w*_1_, *w*_2_) are the sizes of the basins, representing the relative sizes of different basin of attraction. For example, for a bistable system, the probability distribution takes the form: *P*(*x*, *t*) = *w*_1_*P*^*a*^(*x*) + *w*_2_*P*^*b*^(*x*), here *w*_1_ + *w*_2_ = 1. We determine the weights *w*_*i*_ by giving a large number of random initial conditions to the ODEs to be solved, and then collect the statistics from all of these different solutions. For example, for a bistable system, if 10% of the random initial conditions lead to the first steady state, and 90% of the random initial conditions lead to the second steady state, then the weight *w*_1_ for the first basin is 0.1 and *w*_2_ for the second basin is 0.9.

From the mean field approximation, we can extend this formulation to the multidimensional case by assuming that the total probability is the produce of each individual probability for each variable. Finally, we can construct the potential landscape by: *U*(*x*) = −*lnP*_*ss*_(*x*), with *P*_*ss*_ representing the steady-state probability distribution.

To determine how the stable states correspond to different cell states, we first obtain the steady-state expression for different genes from the deterministic ODEs, Eqs. (1–3), and then identify the biological cell states with these steady states, naming them according to their relative gene expression. Based on the cancer biology literature, the genes and miRNAs in Fig. 1 can be classified as either pro-metastatic (including ZEB, SNAIL, OCT4, LIN28, and BACH1), or anti-metastatic (including RKIP, Let7, miR200, miR34, and miR145). Taking the tetrastable landscape (Fig. 2) as an example, we have listed the steady-state gene expression values for 10 different genes for the four stable steady states, individually (Table S5). In this ten-dimensional gene expression state space, the A state (second column in Table S5) has lower relative expression values for all five pro-metastatic genes and high relative expression for all five anti-metastatic genes. Thus, we define this state as the A (anti-metastatic) state. On the contrary, the M state (last column in Table S5) has higher relative expression values for all five pro-metastatic genes and lower relative expression for all five anti-metastatic genes. Therefore, we define this state as the M (metastatic) state. In the middle, the I1 state has higher relative expression for EMT marker genes (such as ZEB and SNAIL) and lower relative expression for metastasis marker gene (such as BACH1), so we defined I1 as an EMT-like state. Similarly, we identified another intermediate state I2.

To validate the self-consistent approximation method, previously we have compared the results from the self-consistent approximation and Langevin dynamics for a simple two-dimensional model.^24^ There we showed that for a simple “mutual repression with self-activation” model, the landscapes from the self-consistent approximation preserve similar global properties (number of attractors, relative stability of the basins of attraction) as the Langevin dynamics method.^24^

### MAPs and optimization of transition actions

Within the cell, there exists intrinsic noise arising from statistical fluctuations of the finite number of molecules, and external noise originating from highly dynamical and inhomogeneous environments, which can be critical to the dynamics of the system.^67–69^ Because the number of cancer cells and immune cells are usually huge, we consider the external noise as the major noise source of the cancer-immunity interplay system. A dynamical system in the fluctuating environments can be described by: $\mathop {{\bf{x}}}\limits^ \cdot = {\bf{F}}\left( {\bf{x}} \right) + \zeta.$ Here, **x** = (*x*_1_(*t*), *x*_2_(*t*),...,*x*_10_(*t*)) represents the vector of the relative gene expressions. **F**(**x**) is the vector for the driving force of chemical reaction. *ζ* is Gaussian noise term whose autocorrelation function is <*ζ*_*i*_(**x**, *t*)*ζ*_*j*_(**x**, 0)> = 2*Dδ*(*t*), and *D* is diffusion coefficient matrix.

Following the approaches^26,52,53,70,71^ based on the Wentzell–Freidlin theory,^72^ the most probable transition path from one attractor *i* at time 0 to attractor *j* at time T, $\phi _{ij}^ \ast (t),t\, \in \,[0,T]$, can be acquired by minimizing the action functional over all possible paths:

7 $$S_T[ {\phi _{ij}} ] = \frac{1}{2}{\int}_0^T | {\mathop {\phi }\nolimits^ \cdot _{ij} - {\mathbf{F}}({\phi _{ij}})} |^2{\mathrm {d}}t.$$

This optimal path is called MAP. We calculated MAPs numerically by applying minimum action methods used in refs. ^52,53^. In our case, *T* is set to be 20 and we verified that larger values of *T* would not improve accuracy of simulations significantly.

To predict the optimal combination of therapeutic targets, we applied the approach of optimizing transition actions to the cancer metastasis model.^52^ Here, the purpose is to predict therapeutic targets (26 parameters characterizing the regulatory strengths among genes) that can induce the transition from M state to A state. Specifically, the optimization process is to minimize the difference between the transition action from M to A and the transition action from A to M, (Δ*S* = *S*_*M*−>*A*_ − *S*_*A*−>*M*_), by tuning each of the 26 parameters. We used the Adaptive Minimum Action Method^53^ to find the minimum of the transition actions, and the matlab fuction “fmincon” was used to perform the minimizations.

### Data availability

The data and matlab codes that support the findings of this study are available from the corresponding author upon reasonable request.

## Electronic supplementary material

Supplementary Methods and Resutls

## Electronic supplementary material

**Supplementary information** accompanies the paper on the *npj Systems Biology and Applications* website (10.1038/s41540-018-0068-x).

## References

[1] Brabletz T, Lyden D, Steeg PS, Werb Z (2013). Roadblocks to translational advances on metastasis research. Nat. Med..

[2] Nieto MA (2011). The ins and outs of the epithelial to mesenchymal transition in health and disease. Annu. Rev. Cell Dev. Biol..

[3] Thiery JP, Acloque H, Huang RY, Nieto MA (2009). Epithelial-mesenchymal transitions in development and disease. Cell.

[4] Nakaya Y, Sheng G (2013). Emt in developmental morphogenesis. Cancer Lett..

[5] Jia D, Jolly MK, Kulkarni P, Levine H (2017). Phenotypic plasticity and cell fate decisions in cancer: insights from dynamical systems theory. Cancers.

[6] Heerboth S (2015). Emt and tumor metastasis. Clin. Transl. Med..

[7] Lu M, Jolly H, Levine H, Onuchic J, Ben-Jacob E (2013). Microrna-based regulation of epithelial-hybrid-mesenchymal fate determination. Proc. Natl Acad. Sci. USA.

[8] Zhang J (2014). Tgf-b-induced epithelial-to-mesenchymal transition proceeds through stepwise activation of multiple feedback loops. Sci. Signal.

[9] Lee J (2014). Network of mutually repressive metastasis regulators can promote cell heterogeneity and metastatic transitions. Proc. Natl Acad. Sci. USA.

[10] Ferrell JE (2012). Bistability, bifurcations, and waddington’s epigenetic landscape. Curr. Biol..

[11] Frauenfelder H, Sligar SG, Wolynes PG (1991). The energy landscapes and motions of proteins. Science.

[12] Qian H (2012). Cooperativity in cellular biochemical processes: noise-enhanced sensitivity, fluctuating enzyme, bistability with nonlinear feedback, and other mechanisms for sigmoidal responses. Annu. Rev. Biophys..

[13] Wang J (2015). Landscape and flux theory of non-equilibrium dynamical systems with application to biology. Adv. Phys..

[14] Wang J, Xu L, Wang EK (2008). Potential landscape and flux framework of non-equilibrium networks: robustness, dissipation and coherence of biochemical oscillations. Proc. Natl Acad. Sci. USA.

[15] Waddington CH (1957). The Strategy of the Genes: A Discussion of Some Aspects of Theoretical Biology.

[16] Wang J, Zhang K, Xu L, Wang EK (2011). Quantifying the waddington landscape and biological paths for development and differentiation. Proc. Natl Acad. Sci. USA.

[17] Wang J, Xu L, Wang EK, Huang S (2010). The potential landscape of genetic circuits imposes the arrow of time in stem cell differentiation. Biophys. J..

[18] Liao C, Lu T (2013). A minimal transcriptional controlling network of regulatory T cell development. J. Phys. Chem. B.

[19] Lv C, Li X, Li F, Li T (2015). Energy landscape reveals that the budding yeast cell cycle is a robust and adaptive multi-stage process. PLoS Comput. Biol..

[20] Li C, Wang J (2014). Landscape and flux reveal a new global view and physical quantification of mammalian cell cycle. Proc. Natl Acad. Sci. USA.

[21] Lu M, Onuchic J, Ben-Jacob E (2014). Construction of an effective landscape for multistate genetic switches. Phys. Rev. Lett..

[22] Ge H, Qian H (2012). Landscapes of non-gradient dynamics without detailed balance: stable limit cycles and multiple attractors. Chaos.

[23] Feng H, Han B, Wang J (2011). Adiabatic and non-adiabatic non-equilibrium stochastic dynamics of single regulating genes. J. Phys. Chem. B.

[24] Li C, Wang J (2013). Quantifying cell fate decisions for differentiation and reprogramming of a human stem cell network: Landscape and biological paths. PLoS Comput. Biol..

[25] Li C, Wang J (2013). Quantifying waddington landscapes and paths of non-adiabatic cell fate decisions for differentiation, reprogramming and transdifferentiation. J. R. Soc. Interface.

[26] Chen C (2014). Mathematical models of the transitions between endocrine therapy responsive and resistant states in breast cancer. J. R. Soc. Interface.

[27] Li C, Wang J (2014). Quantifying the underlying landscape and paths of cancer. J. R. Soc. Interface.

[28] Li C, Wang J (2015). Quantifying the landscape for development and cancer from a core cancer stem cell circuit. Cancer Res..

[29] Huang S (2013). Genetic and non-genetic instability in tumor progression: link between the fitness landscape and the epigenetic landscape of cancer cells. Cancer Metastasis Rev..

[30] Li C, Hong T, Nie Q (2016). Quantifying the landscape and kinetic paths for epithelial–mesenchymal transition from a core circuit. Phys. Chem. Chem. Phys..

[31] Li C (2017). Identifying the optimal anticancer targets from the landscape of a cancer–immunity interaction network. Phys. Chem. Chem. Phys..

[32] Xu L, Zhang K, Wang J (2014). Exploring the mechanisms of differentiation, dedifferentiation, reprogramming and transdifferentiation. PLoS ONE.

[33] Ao P, Galas D, Hood L, Zhu X (2008). Cancer as robust intrinsic state of endogenous molecular-cellular network shaped by evolution. Med. Hypotheses.

[34] Li S, Zhu X, Liu B, Wang G, Ao P (2015). Endogenous molecular network reveals two mechanisms of heterogeneity within gastric cancer. Oncotarget.

[35] Ge H, Qian H (2016). Mesoscopic kinetic basis of macroscopic chemical thermodynamics: a mathematical theory. Phys. Rev. E.

[36] Huang S, Li F, Zhou JX, Qian H (2017). Processes on the emergent landscapes of biochemical reaction networks and heterogeneous cell population dynamics: differentiation in living matters. J. R. Soc. Interface.

[37] Ge H, Qian H (2017). Mathematical formalism of nonequilibrium thermodynamics for nonlinear chemical reaction systems with general rate law. J. Stat. Phys..

[38] Lu M (2014). Toward decoding the principles of cancer metastasis circuits. Cancer Res..

[39] Jolly MK (2014). Towards elucidating the connection between epithelial–mesenchymal transitions and stemness. J. R. Soc. Interface.

[40] Wang J, Zhang K, Wang EK (2010). Kinetic paths, time scale, and underlying landscapes: a path integral framework to study global natures of nonequilibrium systems and networks. J. Chem. Phys..

[41] Wang J, Li C, Wang EK (2010). Potential and flux landscapes quantify the stability and robustness of budding yeast cell cycle network. Proc. Natl Acad. Sci. USA.

[42] Kauffman S (1971). Differentiation of malignant to benign cells. J. Theor. Biol..

[43] Hanahan D, Weinberg RA (2011). Hallmarks of cancer: the next generation. Cell.

[44] Hanahan D, Weinberg RA (2000). The hallmarks of cancer. Cell.

[45] Huang S, Ernberg I, Kauffman S (2009). Cancer attractors: a systems view of tumors from a gene network dynamics and developmental perspective. Semin. Cell Dev. Biol..

[46] Creixell P, Schoof EM, Erler JT, Linding R (2012). Navigating cancer network attractors for tumorspecific therapy. Nat. Biotechnol..

[47] Davies P, Lineweaver C (2011). Cancer tumors as metazoa 1.0: tapping genes of ancient ancestors. Phys. Biol..

[48] Jin H (2010). Snail is critical for tumor growth and metastasis of ovarian carcinoma. Int. J. Cancer.

[49] Liang Y (2012). Transcriptional network analysis identifies bach1 as a master regulator of breast cancer bone metastasis. J. Biol. Chem..

[50] Sachdeva M, Mo YY (2010). mir-145-mediated suppression of cell growth, invasion and metastasis. Am. J. Transl. Res..

[51] Hu X (2013). The heterochronic microrna let-7 inhibits cell motility by regulating the genes in the actin cytoskeleton pathway in breast cancer. Mol. Cancer Res..

[52] Wells DK, Kath WL, Motter AE (2015). Control of stochastic and induced switching in biophysical networks. Phys. Rev. X.

[53] Zhou X (2008). Adaptive minimum action method for the study of rare events. J. Chem. Phys..

[54] Marusyk A, Almendro V, Polyak K (2012). Intra-tumour heterogeneity: a looking glass for cancer?. Nat. Rev. Cancer.

[55] Lawson DA (2015). Single-cell analysis reveals a stem-cell program in human metastatic breast cancer cells. Nature.

[56] Petropoulos S (2016). Single-cell RNA-seq reveals lineage and x chromosome dynamics in human preimplantation embryos. Cell.

[57] Wu F, Su RQ, Lai YC, Wang X (2017). Engineering of a synthetic quadrastable gene network to approach waddington landscape and cell fate determination. eLife.

[58] Kalsbeek A, Merrow M, Roenneberg T, Foster R (2012). Suprachiasmatic nucleus: cellular clocks and networks. Neurobiol. Circadian Timing.

[59] Tetta C, Ghigo E, Silengo L, Deregibus MC, Camussi G (2013). Extracellular vesicles as an emerging mechanism of cell-to-cell communication. Endocrine.

[60] Skog J (2008). Glioblastoma microvesicles transport RNA and proteins that promote tumour growth and provide diagnostic biomarkers. Nat. Cell Biol..

[61] Sasai M, Wolynes P (2003). Stochastic gene expression as a many-body problem. Proc. Natl Acad. Sci. USA.

[62] Zhang B, Wolynes PG (2014). Stem cell differentiation as a many-body problem. Proc. Natl Acad. Sci. USA.

[63] Anderson DF, Craciun G, Kurtz TG (2010). Product-form stationary distributions for deficiency zero chemical reaction networks. Bull. Math. Biol..

[64] Hu, G. *Stochastic Forces and Nonlinear Systems* (Shanghai Scientific and Technological Education Press, Shanghai, 1994).

[65] Van Kampen, N. G. *Stochastic Processes in Chemistry and Physics* (North Holland, Amsterdam, 1992).

[66] Vellela M, Qian H (2009). Stochastic dynamics and non-equilibrium thermodynamics of a bistable chemical system: the schlogl model revisited. J. R. Soc. Interface.

[67] Swain PS, Elowitz MB, Siggia ED (2002). Intrinsic and extrinsic contributions to stochasticity in gene expression. Proc. Natl Acad. Sci. USA.

[68] Kaern M, Elston TC, Blake WJ, Collins JJ (2005). Stochasticity in gene expression: from theories to phenotypes. Nat. Rev. Genet..

[69] Thattai M, Van OA (2001). Intrinsic noise in gene regulatory networks. Proc. Natl Acad. Sci. USA.

[70] Weinan E, Ren W, Vanden-Eijnden E (2004). Minimum action method for the study of rare events. Commun. Pure Appl. Math..

[71] Heymann M, Vanden-Eijnden E (2008). The geometric minimum action method: a least action principle on the space of curves. Commun. Pure Appl. Math..

[72] Freidlin M, Weber M (2004). Random perturbations of dynamical systems and diffusion processes with conservation laws. Probab. Theory Relat. Fields.

