# Supplementary material for: A landscape view on the interplay between EMT and cancer metastasis
Source: NPJ Syst Biol Appl. 2018 Aug 23;4:34. doi: 10.1038/s41540-018-0068-x (PMC6107626; doi:10.1038/s41540-018-0068-x)
Supplement: Supplementary file 1 — Supplementary Methods and Resutls [file 41540_2018_68_MOESM1_ESM.pdf]

Supporting Information for: A landscape view on the interplay between EMT and cancer metastasis

Chunhe Li, Gabor Balazsi

Parameter setting for the models

We determined the parameter values in our models through the following steps:

1). We extracted parameter values from previous studies on gene regulatory networks [1–3]. For example, the Hill coefficient usually reflects the degree of the cooperativity. So, we used the numbers of binding sites for the interaction between microRNAs and proteins to constrain the choice of Hill coefficients (Table S1). In the cases without experimental support, we chose a Hill coefficient n = 4 to represent high cooperativity often observed in gene regulations.

2). To reduce the complexity of the model, and also due to the lack of quantitative experimental data, we set most of the parameters uniformly (i.e. we assumed that the different proteins or RNA have similar synthesis and/or degradation rates unless we found related experimental evidences showing otherwise). For example, we set all the degradation rates to k = 1, most basal synthesis rates to a = 0.2. Of note, we used non-dimensional parameter values in our models to enable mathematical simplicity and to circumvent the lack of extensive experiment data for determining the precise value of all parameters.

3). We set parameter values to satisfy certain biological constrains, including the ability to generate steady state solutions as well as generating multistability, since our purpose is to explore the cell fate decision process for antimetastatic cells, EMT cells, and metastatic cells. To explain the appearance of these different cell types, we believe that it is reasonable to presume the presence of multistability in the models.

4). We performed a global sensitivity analysis to all the parameters in the model (Fig. 5), which supports the robustness of current parameter choices for our models.

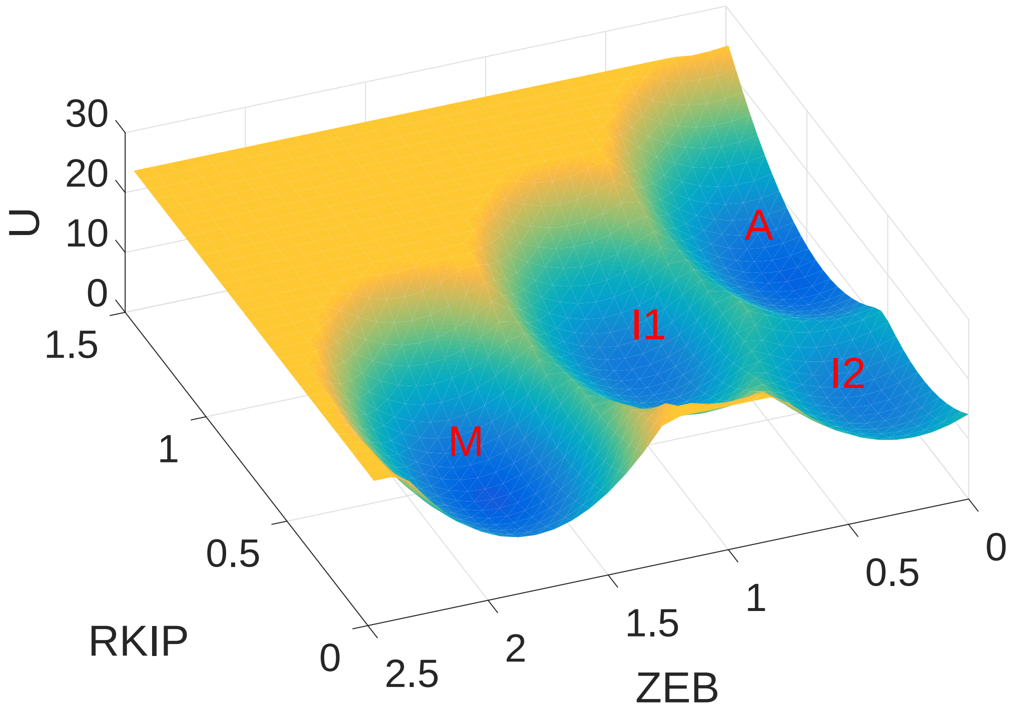

Figure S1. Landscape using RKIP and ZEB as the coordinates. N: normal state; M: metastasis state; I: intermediate states

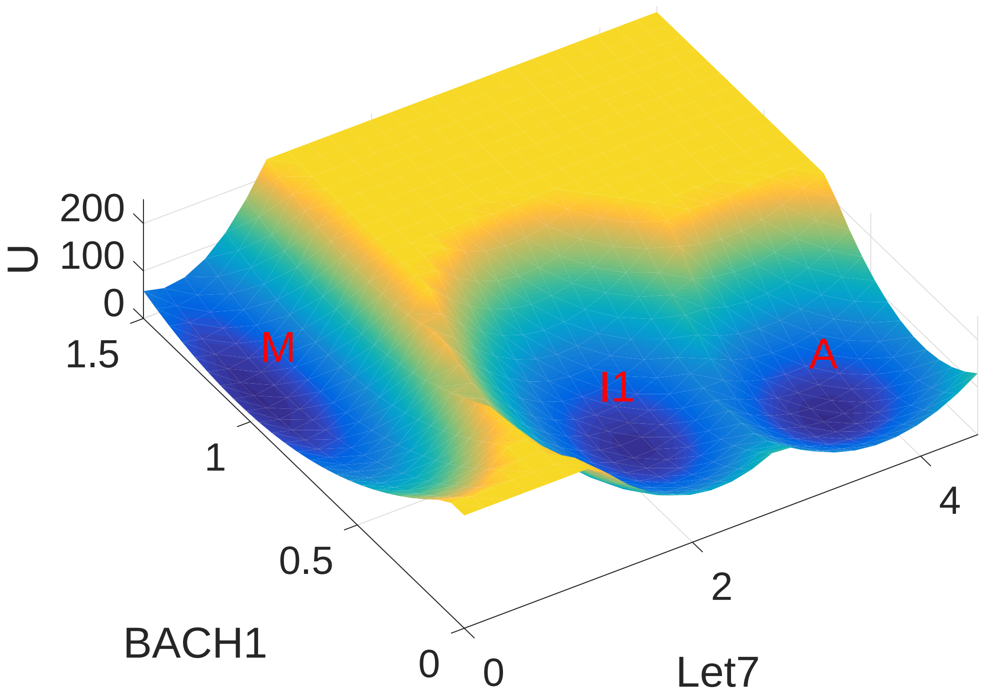

Figure S2. Landscape using Let7 and BACH1 as the coordinates. N: normal state; M: metastasis state; I: intermediate states

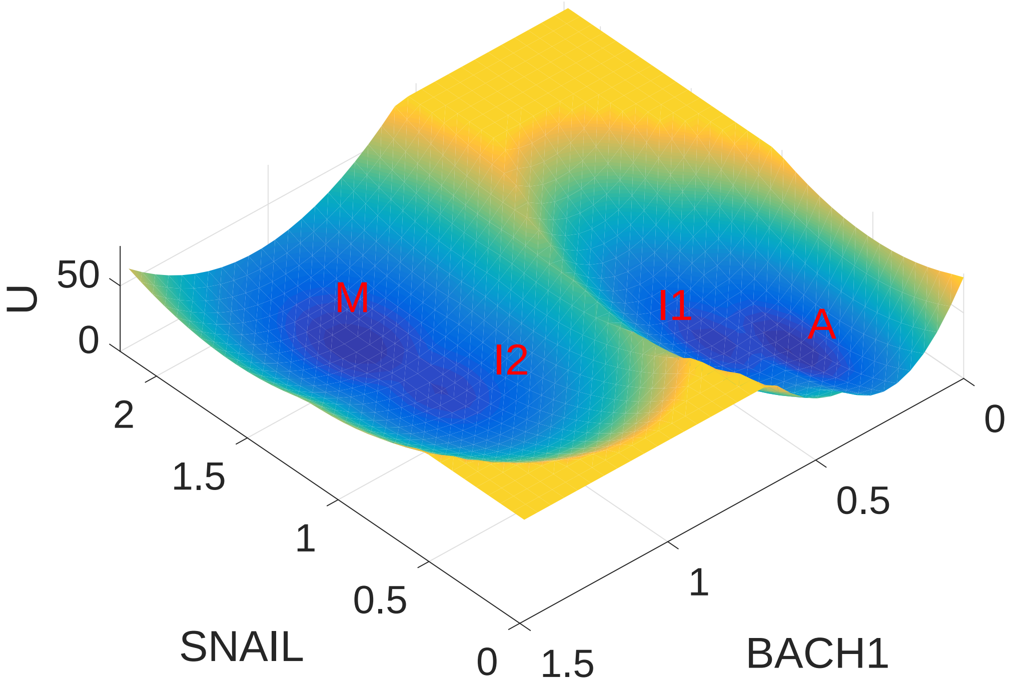

Figure S3. Landscape using SNAIL and BACH1 as the coordinates. N: normal state; M: metastasis state; I: intermediate states

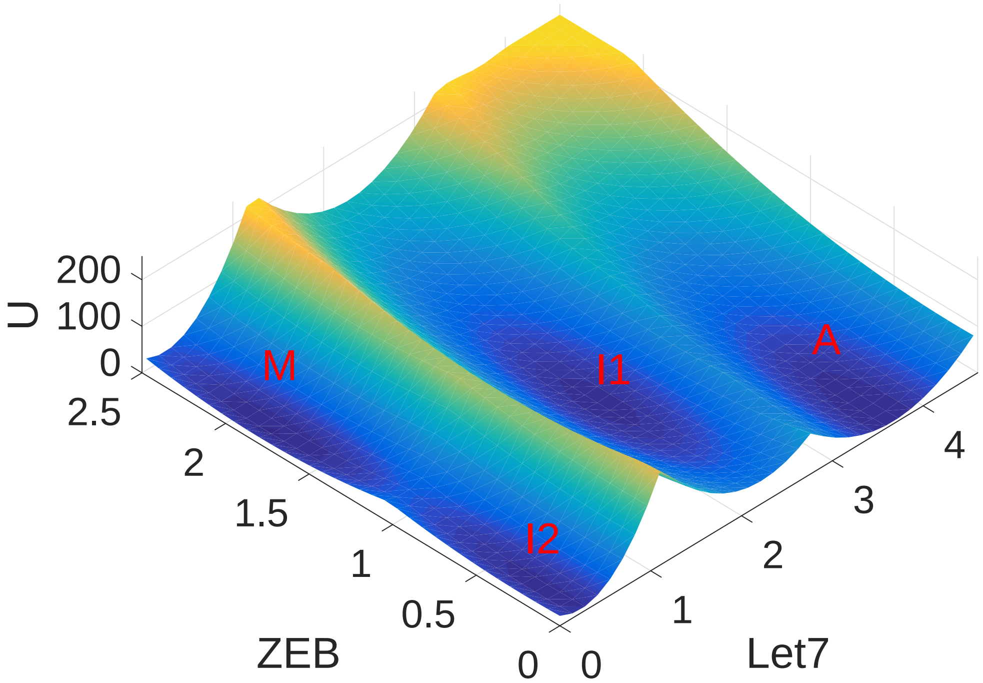

Figure S4. Landscape using Let7 and ZEB as the coordinates. N: normal state; M: metastasis state; I: intermediate states

Table S1. Definitions and values for parameters used in the metastasis model. The numbers of binding sites for the interaction between microRNAs and proteins are used to constrain the value of Hill coefficients. For the interaction with no experimental evidences, the default Hill coefficient value is used.

Symbol	Definition	Value	References
n	Default Hill coefficient	4	
ns	Default Hill coefficient for self-activation	2	
S	threshold for Hill function	0.5	
b	repression constant	0.8	
a	activation constant	0.2	
sa1	self-activation constant for oncogene	0.2	
sa2	self-activation constant for tumor repressor	0.8	
k	degradation constant	1	
k_{LB}	binding rate between Let7 and BACH1	4	[4]
S_R	activation threshold for the regulation of RKIP on Let7	0.5	[4]
S_B	threshold for self-repression of BACH1	1	[4]
$G0_B$	basal synthesis rate of BACH1	0.1	[4]
G_B	Maximum synthesis rate of BACH1	4	[4]
G_L	activation constant for the regulation of RKIP on Let7	7	[4]
nr	Hill coefficient for the regulation of RKIP on Let7	5	[4]
nb	Hill coefficient for self-repression of BACH1	3	[4]
n_{BR}	Hill coefficient for repression of BACH1 to RKIP	1	[4]
n_{SS}	Hill coefficient for self-repression of SNAIL	1	[5]
n_{SZ}	Hill coefficient for activation of SNAIL to ZEB	1	[5]
n_{Sm200}	Hill coefficient for repression of SNAIL to miR200	1	[5]
n_{Sm34}	Hill coefficient for repression of SNAIL to miR34	1	[5]
n_{Zm200}	Hill coefficient for repression of ZEB to miR200	3	[5]
n_{Zm34}	Hill coefficient for repression of ZEB to miR34	2	[5]
n_{m200Z}	Hill coefficient for repression of miR200 to ZEB	6	[5]
n_{m34S}	Hill coefficient for repression of miR34 to SNAIL	2	[5]

Table S2. Regulation matrix M. The element $M(j,i)$ (the jth row and the ith column of the matrix M) represents the regulation type from node j to node i. 1 represents activation, −1 represents repression, and 0 represents no interaction.

Gene	SNAIL	ZEB	OCT4	miR200	miR34	miR145	Let7	RKIP	LIN28	BACH1
	1	2	3	4	5	6	7	8	9	10
1	−1	1	0	−1	−1	0	0	−1	0	0
2	0	1	0	−1	−1	−1	0	0	0	0
3	0	0	1	1	0	−1	0	0	0	0
4	0	−1	0	0	0	0	0	0	0	0
5	−1	0	0	0	0	0	0	0	0	0
6	0	−1	−1	0	0	0	0	0	0	0
7	−1	0	0	0	0	0	1	0	−1	−1
8	0	0	0	0	0	0	1	0	0	0
9	0	0	1	0	0	0	−1	0	1	0
10	0	0	0	0	0	0	0	−1	0	−1

Table S3. Target ID (regulation link ID) and corresponding links (18 activation regulations and 8 inhibition regulations), as well as related references. Here, $->$ represent activation, and $-|$ represent inhibition.

Target ID	regulations	References
1	SNAIL −| SNAIL	[5]
2	miR34 −| SNAIL	[5]
3	Let7 −| SNAIL	[5]
4	SNAIL − > ZEB	[5]
5	ZEB − > ZEB	[5]
6	miR200 −| ZEB	[5]
7	miR145 −| ZEB	[5]
8	OCT4− >OCT4	[5]
9	miR145−|OCT4	[5]
10	LIN28− >OCT4	[5]
11	SNAIL−|miR200	[5]
12	ZEB−|miR200	[5]
13	OCT4− >miR200	[5, 6]
14	SNAIL−|miR34	[5]
15	ZEB−|miR34	[5]
16	ZEB−|miR145	[5]
17	OCT4 −|miR145	[5]
18	Let7 − >Let7	[5, 7]
19	RKIP− >Let7	[4]
20	LIN28 −|Let7	[5, 8]
21	SNAIL −|RKIP	[5]
22	BACH1 −|RKIP	[4]
23	Let7 −|LIN28	[5]
24	LIN28 − >LIN28	[5]
25	Let7 −|BACH1	[4]
26	BACH1 −|BACH1	[4]

Table S4. Metastasis-suppressing interventions identified from sensitivity analysis (Fig. 5 in main text). Each regulation is represented by a target ID (Table S3). Sensitivity is defined as the change of transition actions from M to A attractors as each parameter (regulation strength) changes. Here, the regulations are sorted by sensitivity and the top 10 regulations are showed.

Sensitivity	Target ID
0.409	9
0.201	17
0.194	22
0.128	7
0.122	11
0.107	2
0.090	8
0.086	26
0.079	19
0.079	4

Table S5. Steady states gene expression values for tetrastable landscape (corresponding to Fig. 2 in main text). A: antimetastatic state, M: metastatic state, I1, I2: intermediate state.

Genes	A	I1	I2	M
SNAIL	0.5208	0.7317	1.2232	1.6333
ZEB	0.1288	0.9637	0.2301	1.8078
OCT4	0.0077	0.0908	0.2301	1.1545
miR200	1.1784	0.4230	0.9697	0.3973
miR34	1.1421	0.4944	0.8923	0.2444
miR145	1.5965	0.8532	1.5314	0.0318
Let7	3.6501	2.1158	0.0352	0.0246
RKIP	0.8948	0.5844	0.2492	0.2323
LIN28	0.0003	0.0025	0.9571	0.9571
BACH1	0.2585	0.4066	1.2586	1.2754

References

1. Li C, Wang J (2013) Quantifying cell fate decisions for differentiation and reprogramming of a human stem cell network: Landscape and biological paths. PLoS Comput Biol 9: e1003165.

2. Li C, Wang J (2014) Quantifying the underlying landscape and paths of cancer. J R Soc Interface 10: 20140774.

3. Huang S, Guo Y, May G, Enver T (2007) Bifurcation dynamics of cell fate decision lineage-commitment in bipotent progenitor cells. Dev Biol 305: 695-713.

4. Lee J, Lee J, Farquhar KS, Yun J, Frankenberger CA, et al. (2014) Network of mutually repressive metastasis regulators can promote cell heterogeneity and metastatic transitions. Proceedings of the National Academy of Sciences 111: E364–E373.

5. Lu M, Jolly MK, Ben-Jacob E, et al. (2014) Toward decoding the principles of cancer metastasis circuits. Cancer research 74: 4574–4587.

6. Wang G, Guo X, Hong W, Liu Q, Wei T, et al. (2013) Critical regulation of mir-200/zeb2 pathway in oct4/sox2-induced mesenchymal-to-epithelial transition and induced pluripotent stem cell generation. Proceedings of the National Academy of Sciences 110: 2858–2863.

7. Zisoulis DG, Kai ZS, Chang RK, Pasquinelli AE (2012) Autoregulation of microrna biogenesis by let-7 and argonaute. Nature 486: 541–544.

8. Stefani G, Chen X, Zhao H, Slack FJ (2015) A novel mechanism of lin-28 regulation of let-7 microrna expression revealed by in vivo hits-clip in c. elegans. Rna 21: 985–996.
